# Presence of CP4-EPSPS Component in Roundup Ready Soybean-Derived Food Products

**DOI:** 10.3390/ijms13021919

**Published:** 2012-02-10

**Authors:** Honghong Wu, Yu Zhang, Changqing Zhu, Xiao Xiao, Xinghu Zhou, Sheng Xu, Wenbiao Shen, Ming Huang

**Affiliations:** 1 College of Life Sciences, Nanjing Agricultural University, Nanjing 210095, China; E-Mails: 2008116134@njau.edu.cn (H.W.); 2009116133@njau.edu.cn (X.X.); 2008216034@njau.edu.cn (S.X.); 2 College of Food Sciences, National Center of Meat Quality and Safety Control, Nanjing Agricultural University, Nanjing 210095, China; E-Mail: zxh399380548@sina.com; 3 Division of Chemistry and Biological Chemistry, School of Physical and Mathematical Sciences, Nanyang Technological University, Singapore 637371, Singapore; E-Mail: zh0010yu@e.ntu.edu.sg; 4 Jiangsu Entry-Exit Inspection and Quarantine Bureau, Nanjing 210001, China; E-Mail: changqing.zhu@126.com

**Keywords:** foodstuffs, Roundup Ready soybean, soya protein concentrates, traceability of CP4-EPSPS, western blot

## Abstract

With the widespread use of Roundup Ready soya (event 40-3-2) (RRS), the traceability of transgenic components, especially protein residues, in different soya-related foodstuffs has become an important issue. In this report, transgenic components in commercial soya (including RRS) protein concentrates were firstly detected by using polymerase chain reaction (PCR) and western blot. The results illustrated the different degradation patterns of the *cp4-epsps* gene and corresponding protein in RRS-derived protein concentrates. Furthermore, western blot was applied to investigate the single factor of food processing and the matrix on the disintegration of CP4-EPSPS protein in RRS powder and soya-derived foodstuffs, and trace the degradation patterns during the food production chain. Our results suggested that the exogenous full length of CP4-EPSPS protein in RRS powder was distinctively sensitive to various heat treatments, including heat, microwave and autoclave (especially), and only one degradation fragment (23.4 kD) of CP4-EPSPS protein was apparently observed when autoclaving was applied. By tracing the protein degradation during RRS-related products, including tofu, tou-kan, and bean curd sheets, however, four degradation fragments (42.9, 38.2, 32.2 and 23.4 kD) were displayed, suggesting that both boiling and bittern adding procedures might have extensive effects on CP4-EPSPS protein degradation. Our results thus confirmed that the distinctive residues of the CP4-EPSPS component could be traced in RRS-related foodstuffs.

## 1. Introduction

Roundup Ready soybean (event 40-3-2) (RRS) is one of the major genetically modified crop species. It is equivalent to traditional soya and the presence of the 5-enolpyruvylshikimate-3-phosphate synthesis from *Agrobacterium* sp. strain CP4 (CP4-EPSPS) gene and the corresponding protein. RRS has been authorized for the market in many countries and trading areas since the approval of its cultivation in some countries [1]. Considering the significant importance of soya products in human nutrition, RRS is firmly established in the human diet and has gained great popularity in the global market, especially in China [2]. For example, the main food products and food ingredients derived from soybean in China are soya-derived products, such as tofu, tou-kan and bean curd sheets, as well as soya protein concentrates or isolates used in the food industry. However, the possible risk of transgenic components of RRS in related food products has become an important issue. To ensure food safety control, all the foodstuffs derived from genetically modified technology must undergo a comprehensive evaluation before entering the market. Such assessment is of legal importance as part of the regulatory approval [3].

Until now, most research on RRS assessments has been mainly carried out to evaluate the transgenic components in soya-related foodstuffs or trace CP4-EPSPS residue during the production chain of certain food products [4–6]. However, little is known about the transgenic components in soya protein concentrates even though they are the main food additives in the food production [7]. On the other hand, research has tended to focus on a DNA-based approach for detecting the degradation of transgenic component and the post-market traceability [6,8]. PCR and its derived approaches have been routinely applied during the safety assessment of RRS and the corresponding food products [4,9,10]. In contrast, few have applied the protein-based approach as it is more time-consuming, with a high cost for antibody preparation and protein sequencing.

However, detection of exogenous protein is of great concern as it is linked to potential risks such as allergenicity, toxicity and dietary risks. Accordingly, the denaturation and degradation during food processing are important, assuming introduced protein(s) may not lose functional activity during the processing of raw grain into food [3]. Current protein-based detection involves mainly the application of immunological methods such as western blot and the enzyme-linked immunosorbent assays (ELISA). Between them, western blot is more favored in situations where the tested protein is difficult to extract. This method can evaluate and trace the degradation characteristics of the target protein [11].

Considering the deduced risks of exogenous protein, it is crucial to reveal the effect of food processing on exogenous protein and detect its residue more accurately. Meanwhile, traceability along the food production chain would determine whether the transgenic component in foodstuffs or corresponding middle products are from genetically modified (GM) sources or due to the GM contamination during manufacturing [6]. Thus, the determination of the stability and/or stable fragments during the above process is of great significance.

In our study, we performed semi-quantitative PCR and western blot to study the different disintegration modes of *CP4-EPSPS* gene and protein in the commercial soya (including RRS) protein concentrates, since soy protein concentrates may be used as functional ingredients in the food industry. Interestingly, we discovered discrepancies in the degradation profiles of *cp4-epsps* gene and the corresponding protein among certain protein concentrate samples purchased from different sources. For further confirmation, western blot was applied to evaluate protein degradation of RRS powder responding to a particular factor which might occur in food processing, such as heating, microwave and autoclave. Middle- and end-products during the manufacturing processes of tofu, such as raw soya milk, boiled soya milk, bean dregs, tofu jelly and tofu, tou-kan and bean curd sheets, were examined and compared, to reveal the effect of the different food matrix on the changes of CP4-EPSPS protein and to trace the protein degradation patterns along the processing chain. The aim of the study was to investigate the effects of a single factor and the food matrix during the manufacturing on the degradation of CP4-EPSPS protein, and to reveal the possible residues of transgenic components in soya protein concentrates and RRS-derived foodstuffs. Additionally, we sought to provide more reliable and available evidence of the protein-based detection approach for the traceability of CP4-EPSPS protein in various RRS-derived food products.

## 2. Results and Discussion

### 2.1. Detection of Transgenic Components in Commercial Soya Protein Concentrates

**Figure 1:**
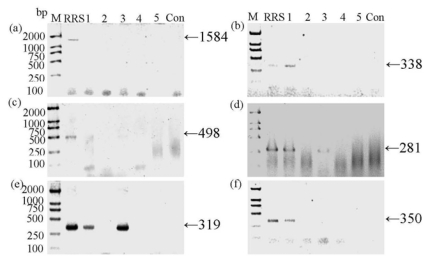
Agarose gel electrophoresis of PCR products of *cp4-epsps* gene fragments in commercial soya protein concentrates purchased from different cities in China. Different primer pairs used were shown in Table 1. Lane M, molecular weight marker (DL 2000™ Marker, TaKaRa); Roundup Ready soya (RRS), unprocessed RRS powder; Lane 1–5, commercial soya protein concentrates, purchased from Anyang (Henan Province, labeled to be RRS protein concentrate), Nantong (Jiangsu Province), Hangzhou (Zhejiang Province), Nanjing (Jiangsu Province) and Anheng (Anhui Province), respectively; unprocessed non-transgenic soya powder (Con). The sizes of the expected PCR amplification products are indicated by the *arrows* on the right.

In our study, commercial soya protein concentrates were purchased from markets in different cities of China, among which only one sample from Anyang (Henan Province) was labeled as RRS protein concentrate. Then, qualitative PCR [12] and western blot were applied to detect the transgenic contents of these samples. For PCR, six primer pairs were used (Table 1). The efficiency of DNA extraction was confirmed by the PCR of *lectin* gene (data not shown). Our results further suggested that exogenous DNA degradation had been taken place during or after the processing of the protein concentrates (Figure 1a) as the band of full-length *cp4-epsps* gene (1584 bp) [13] only showed in the unprocessed RRS sample (RRS), while absent in all the five RRS protein concentrates and the unprocessed non-transgenic soya powder (Con). Meanwhile, fragments with the size of 338, 281, 319 and 350 bp were detected and sequenced in the RRS protein concentrates from Anyang (Figure 1), suggesting the full-length gene had been disintegrated into smaller fragments. Similar results have been presented previously [14], claiming the presence of transgenic soya DNA in RRS powder and RRS-related protein concentrates. In comparison with those of three commercial soya protein concentrates bought from Nantong (Jiangsu Province), Nanjing (Jiangsu Province) and Anheng (Anhui Province), two bands (281 bp and 319 bp), which were also confirmed by sequencing, appeared in the samples from Hangzhou (Zhejiang Province), suggesting the presence of residue in this protein concentrate (Figure 1d,e). A related explanation might be attributed to the fact that the soya protein concentrates purchased from Hangzhou, assuming it was RR-related, displayed a different degradation pattern of *cp4-epsps* caused by other manufacturing technologies of soya protein isolates. Previous studies have already confirmed that different processing procedures led to various *cp4-epsps* DNA degradation patterns in the corresponding food products [15–18].

**Figure 2:**
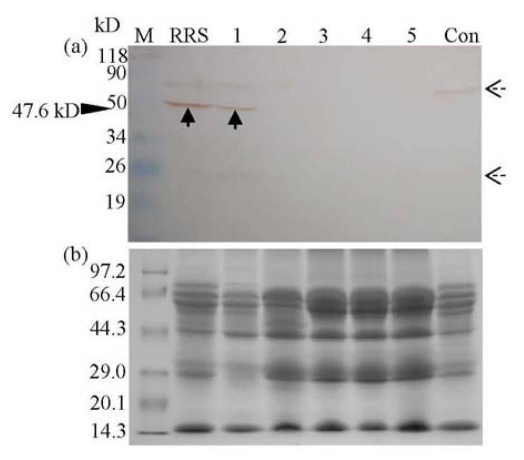
Western blot analysis of the CP4-EPSPS protein in commercial soya protein concentrates purchased from different cities in China. Lane M, protein molecular weight marker (a, prestained protein molecular marker, Fermentas; b, protein molecular weight marker, TaKaRa); Lane RRS, unprocessed RRS powder; Lane 1–5, commercial soya protein concentrates, purchased from Anyang (Henan Province, labeled to be RRS protein concentrate), Nantong (Jiangsu Province), Hangzhou (Zhejiang Province), Nanjing (Jiangsu Province) and Anheng (Anhui Province), respectively; unprocessed non-transgenic soya powder (Con). The antibody used was SC-16 (**a**). Corresponding Coomassie Brilliant Bluestained gels of (**a**) are also provided (**b**). The solid black arrow shows the immunoreactive band corresponding to mature CP4-EPSPS protein (47.6 kD). The dashed black arrows present in the western blot are due to the non-specific staining because both of them existed in the RRS and Con samples.

However, western blot demonstrated distinctive results. Mature CP4-EPSPS protein (47.6 kD) only presented in RRS and its protein concentrate from Anyang (Henan Province) but was absent in non-transgenic soya as well as four other commercial soya protein isolates, confirming the specificity of the antibody we used (Figure 2a). Compared to the PCR results (Figure 1), no mature or degraded protein was detected in the samples from Hangzhou (Zhejiang Province). This distinction could be explained by the hypothesis that the *cp4-epsps* gene and corresponding protein responded differentally to food processing, and the protein, provided that it was RR-related, was degraded either completely to smaller peptides or amino acids, or partially to fragments that could not be detected by the antibody used in our study.

### 2.2. Effect of Single-Factor Treatment on CP4-EPSPS Protein Degradation

It is well known that heating is one of the major ways of processing commercial soya protein concentrates. To investigate the effects of food processing on CP4-EPSPS protein degradation and its traceability along the production chain, three factors, namely heat, microwave and autoclave were examined separately. These factors are all closely involved in food manufacturing, used to produce products like tofu and soya drinks. We also applied unprocessed 100% RRS as the positive control [19].

**Figure 3:**
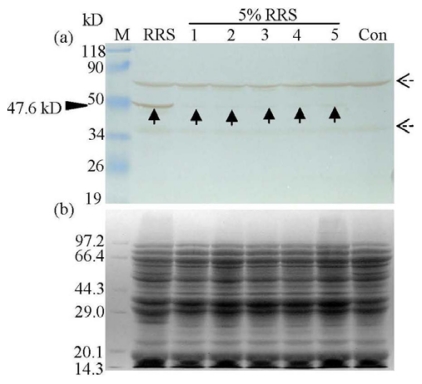
Western blot analysis of the CP4-EPSPS protein in heat processed 5% RRS powder (5% RRS). Lane M, protein molecular weight marker (a, prestained protein molecular marker, Fermentas; b, protein molecular weight marker, TaKaRa); Lane RRS, unprocessed 100% RRS powder; Lane 1, 2, 3, 4, and 5: heat processed 5% RRS powder for 5, 15, 30, 60, and 120 min, respectively; Lane Con, unprocessed non-transgenic soya powder. The antibody used was SC-16 (**a**). Corresponding Coomassie Brilliant Blue-stained gels of (**a**) are also provided (**b**). The solid black arrow shows the immunoreactive band corresponding to mature CP4-EPSPS protein (47.6 kD). The dashed black arrows present in the western blot are due to the non-specific staining because both of them existed in the RRS and Con samples.

For heat treatment, the native CP4-EPSPS protein (47.6 kD) was only absent in unprocessed non-transgenic soya (Con) (Figure 3a). Meanwhile, no significant degradation of native CP4-EPSPS protein and total protein (Figure 3b) were observed in either of the 5% RRS samples upon heat treatment. The results indicated that the native protein may be robust to heat treatment, although the partial degradation of the CP4-EPSPS protein could not be easily ruled out, because the N-terminal of CP4-EPSPS protein is the recognition site of the SC-16 antibody.

**Figure 4:**
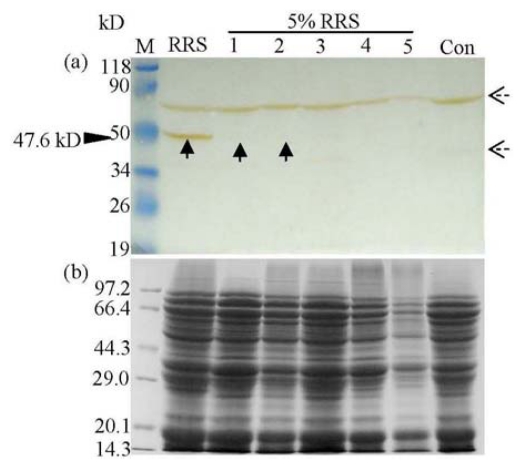
Western blot analysis of the CP4-EPSPS protein in microwave processed 5% RRS powder (5% RRS). Lane M, protein molecular weight marker (a, prestained protein molecular marker, Fermentas; b, protein molecular weight marker, TaKaRa); Lane RRS, unprocessed 100% RRS powder; Lane 1, 2, 3, 4, and 5: microwave processed 5% RRS powder for 1, 5, 10, 20, and 30 min, respectively; Lane Con, unprocessed non-transgenic soya powder. The antibody used was SC-16 (**a**). Corresponding Coomassie Brilliant Bluestained gels of (**a**) are also provided (**b**). The solid black arrow shows the immunoreactive band corresponding to mature CP4-EPSPS protein (47.6 kD). The dashed black arrows present in the western blot are due to the non-specific staining because both of them existed in the RRS and Con samples.

Microwave treatment, simulating dry heating, showed a more intensive effect on the CP4-EPSPS protein, in that when the treatment time was raised to 35 min, the soya powder was totally carbonized. Accordingly, we only examined RRS exposed to microwaves for a maximum of 30 min at the maximum power. The mature band of CP4-EPSPS (47.6 kD) could only be detected in the unprocessed RRS and processed sample for 1 min and 5 min of treatment. When the treatment time was raised to 10 min, native protein could not be detected, confirming the decomposition of the mature protein. In addition, after a treatment of 30 min, degradation of the total protein could be clearly observed on the SDS-PAGE gel (Figure 4b).

**Figure 5:**
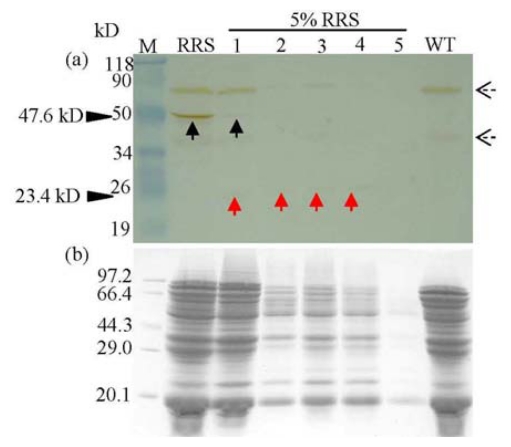
Western blot analysis of the CP4-EPSPS protein in autoclaving processed 5% RRS powder (5% RRS). Lane M, protein molecular weight marker (a, prestained protein molecular marker, Fermentas; b, protein molecular weight marker, TaKaRa); Lane RRS, unprocessed 100% RRS powder; Lane 1, 2, 3, 4, and 5: autoclaving processed 5% RRS powder for 10, 20, 30, 60, and 120 min, respectively; Lane Con, unprocessed non-transgenic soya powder. The antibody used was SC-16 (**a**). Corresponding Coomassie Brilliant Blue-stained gels of (**a**) are also provided (**b**). The solid black arrow shows the immunoreactive band corresponding to mature CP4-EPSPS protein (47.6 kD). The red arrows show the immunoreactive bands corresponding to the possible degradation fragments of the CP4-EPSPS protein (23.4 kD). The dashed black arrows present in the western blot are due to the non-specific staining because both of them existed in RRS and Con samples.

Autoclaving, used to manufacture soya drink, textured vegetable protein, soybean meal, maize instant tortilla, *etc*., generated a more violent effect on protein in contrast to heat and microwave treatment. Mature CP4-EPSPS protein was only detected in the unprocessed soya and the 5% RRS sample that went through with only 10 min of autoclaving treatment. A degradation fragment (23.4 kD) appeared at the time interval from 10 min to 60 min but was absent when the treatment time was raised to 120 min (Figure 5a). The amount of the degradation product (23.4 kD) increased slightly during the time interval, indicating the dramatic impact of such treatment on the CP4-EPSPS protein degradation. Similarly, total protein displayed significant disintegration during the whole treatment period. Based on SDS-PAGE results, it could scarcely be observed when the treatment time reached 120 min (Figure 5b).

Together, our results disclosed that the CP4-EPSPS protein was sensitive to heat, microwave and autoclave, but to different degrees. Microwave and autoclaving exhibit the most extensive impact on the CP4-EPSPS protein and total protein in RRS powder, followed by treatments. Yet, only one degradation fragment with the size of 23.4 kD was observed when autoclaving was applied, which might be also attributable to the suitable recognition site of the antibody. Considering this, we further suggested that the N-terminal amino acid sequence might be more sensitive to these treatments mentioned above. Previous study has claimed that the food processing of soya ingredients would result in the loss of immunochemical recognition [14].

### 2.3. Effect of Food Matrix on CP4-EPSPS Protein Degradation and Traceability during Food Processing

**Figure 6:**
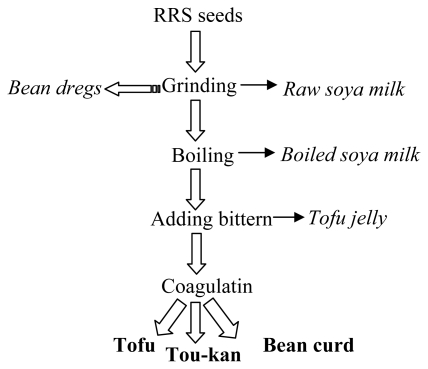
Sampling from the production chain of RRS-derived products.

**Figure 7:**
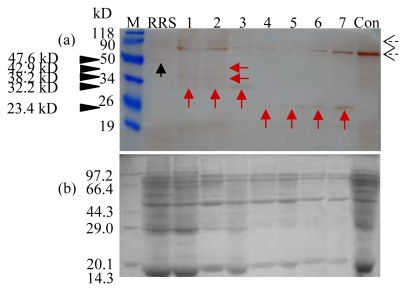
Western blot analysis of the CP4-EPSPS protein in Roundup Ready soybean-derived food products. Lane M, protein molecular weight marker (a, prestained protein molecular weight marker, Fermentas; b, protein molecular weight marker, TaKaRa); Lane RRS, unprocessed 100% RRS powder; Lane 1, raw soya milk; Lane 2, boiled soya milk; Lane 3, bean dregs; Lane 4, tofu jelly; Lane 5, tofu; Lane 6, tou-kan; Lane 7, bean curd sheets; Lane Con, unprocessed non-transgenic soya powder. The antibody used was SC-16 (**a**). Corresponding Coomassie Brilliant Blue-stained gels of (**a**) is also provided (**b**). The solid black and red arrows show the immunoreactive band corresponding to mature and the possible degradation fragments of the CP4-EPSPS protein (47.6; 42.9, 38.2, 32.2 and 23.4 kD). The dashed black arrows present in the western blot are due to the non-specific staining because both of them existed in the RRS and Con samples.

To take the study further, we investigated the effect of the food matrix on the CP4-EPSPS protein degradation and traced the protein along the production chain. Grinding, boiling, bittern adding and coagulating are four critical processes in tofu, tou-kan and bean curd sheets production and the middle-products are raw soya milk, boiled soya milk, bean dregs and tofu jelly (Figure 6). Results in Figure 7a showed that the native CP4-EPSPS protein was only detected in unprocessed RRS and absent in all other samples. Four degradation fragments were displayed, with the molecular weight of 42.9, 38.2, 32.2 and 23.4 kD, respectively. We noticed that the amount of the smallest degradation product (23.4 kD) increased steadily during processing from middle-products tofu jelly to the three end products, suggesting the procedure of bittern adding and coagulating might have a dramatic impact on the CP4-EPSPS protein. Such a fragment was also found when autoclaving was applied (Figure 5a). Autoclaving simulates moist heating under pressure, used to manufacture soy drink, textured vegetable protein, and soya meal. Accordingly, we concluded that the effects of heating up and squeezing involved in the procedures of bittern adding and coagulating might be mimicked by autoclaving, at least partially, thus generating a similar fragment of the CP4-EPSPS protein. Additionally, the total protein in all of the tested samples was largely degraded after the process of boiling (Figure 7b). In contrast with the above results by using a single factor test (Figures 3–5), it was further intimated that the degradation site of the CP4-EPSPS protein might vary in response to the food matrix.

Previous study, applying quantitative PCR (qPCR) detected the *cp4-epsps* degradation during a similar process [4] and suggested the concentration of transgenic component remained stable during manufacturing. Similarly, another study traced the *cp4-epsps* degradation during tofu production, and demonstrated that the procedures of boiling as well as bittern adding did not cause dramatic DNA degradation as a 595-bp could be detected throughout the process [20]. By contrast, a previous report examined the change of gene during soymilk processing produced by RRS, and revealed that thermal treatment had a relatively more dramatic effect on the exogenous gene than on the endogenous gene [21]. However, little study has covered the traceability of the CP4-EPSPS protein along the food manufacturing line. Considering the risks of exogenous protein such as allergenicity and toxicity, it is of great value to detect the exogenous protein and its residue at a realistic and precise value. Moreover, determination of the stability and stable fragments during the processing is of great importance as it will provide sufficient evidence as to whether introduced gene(s)/protein(s) would lose functional activity during processing of raw grain into food [20,22]. In this report, we confirmed the validity of western blot to trace transgenic components and their degradation, since the specifically degraded four fragments of the CP4-EPSPS protein could be clearly observed. Additionally, our traceability along the food production chain might be used as an approach to determine the transgenic components in food products, or in the corresponding middle products produced from RRS sources, or due to the RRS contamination during manufacturing.

## 3. Experimental Section

### 3.1. Plant Materials

Crushed powder of RRS (event 40-3-2), soya standard with RRS concentration of 5% or 100% and non-transgenic soya (Con) were kindly provided by Jiangsu Province Entry-Exit Inspection and Quarantine Bureau, China, and all of them have been certified. For commercial soya protein concentrates analysis, five soybean protein isolate products were purchased separately from markets in different cities in China, namely Anyang (Henan Province), Nantong (Jiangsu Province), Hangzhou (Zhejiang Province), Nanjing (Jiangsu Province) and Anheng (Anhui Province).

### 3.2. Heat Processing Operation

Soya powder (1.0 g) was directly placed in an Eppendorf tube. Heat processing operations (95 °C) in a water bath (DK-BD, Shanghai Yiheng Technology Co., Ltd., Shanghai, China) were carried out for the indicated time intervals. At least three replicates were performed.

### 3.3. Microwave Treatment

Soya powder (3.0 g) was spread homogeneously and heated in a microwave oven (MWO; WP700P21, Galanz, Shunde, China) at 750 W for the indicated times. The MWO exposure was restricted to 30 min as serious charring occurred when subjected to 35 min. All the processing steps were carried out in triplicate.

### 3.4. Autoclaving

Soya powder (3.0 g) was directly autoclaved using an automated high-pressure steam sterilizer (LDZX-30KBS, Shanghai Shenan Medical Instrument Factory, Shanghai, China) at 121 °C and 0.1 MPa for the indicated times. The heating and cooling time was not included. All the processing steps were carried out in triplicate.

### 3.5. Food Matrix

Transgenic RRS was produced into soya-derived food products such as tofu, tou-kan and bean curd sheets. According to critical processing procedures (Figure 6), certain middle products (raw soya milk, boiled soya milk, bean dregs and tofu jelly) and three end products (tofu, tou-kan and bean curd sheets) were taken as samples.

### 3.6. DNA Extraction

DNA was extracted from the samples mentioned above with an identical fresh weight by using a cetyltrimethylammonium bromide (CTAB) method [12].

### 3.7. PCR Reaction

Primer sequences and related information of *cp4-epsps*, were presented in Table 1. The PCR reaction was run as follows: an initial denaturation at 94 °C for 10 min; with 40 cycles at 94 °C for 1 min, annealing (for each primer, annealing temperature was listed in Table 1) for 1 min, 72 °C for 20–100 s (depending on the length of amplicon); and a final extension at 72 °C for 10 min. The amplified fragments were analyzed by 1.5% agarose gel electrophoresis stained with ethidium bromide. Each extract was amplified at least in duplicate assays. Finally, the PCR products were purified with gel extraction using AxyPrep Gel DNA Extraction Kit (Axygen, Hangzhou, China) according to the manufacturer’s protocol. The purified products were then cloned into the pMD19-T vector (TaKaRa) for sequencing (GenScript, Nanjing, China).

### 3.8. Protein Extraction

Total proteins from different samples (0.3 g) were extracted in 1 mL cold TPBS buffer (150 mM NaCl, 8 mM Na_2_HPO_4_·12H_2_O, 2 mM KH_2_PO_4_·H_2_O, 4 mM KCl, 0.05% Tween-20, and 2% SDS, pH 7.4). After shaking for 30 minutes at room temperature, the mixture was centrifuged twice at 20,000 g in a rotor (model Avanti J-25, Beckman) for 15 min each. Total protein concentration was determined by BCA (Bicinchoninic acid) assay (Multisciences Biotechnology Co. Ltd., Hangzhou, China), using bovine serum albumin (BSA) as a standard.

### 3.9. Western Blotting Analysis

The predicted protein product of CP4-EPSPS comprises 527 amino acids with molecular weight of 55.5 kD, which includes a 72 amino acid chloroplastic transit peptide proven previously [13], indicating a mature protein of 47.6 kD following the cleavage site of the transit peptide. In our experiment, one peptide sequence containing the specific amino acids corresponding to the mature CP4-EPSPS sequence positions (S19→R33) for the antigen were obtained by chemical synthesis. The peptide was hydrophilic, surface-oriented, and flexible. Synthetic peptides were purified by HPLC and coupled to keyhole limpet haemacyanin (KLH). The CP4-EPSPS-KLHs were collected and used for producing the rabbit polyclonal antibody (SC-16).

For the western blot analysis, protein (30 μg) or protein extracted from an equal weight of different samples was subjected to SDS-PAGE using a 12.5% acrylamide resolving gel (Mini Protean II System, Bio-Rad, Hertz, UK). Separated proteins were then transferred to polyvinylidene difluoride (PVDF) membranes, and non-specific binding of antibodies was blocked with 5% non-fat dried milk in phosphate-buffered saline (PBS, pH 7.4) overnight at 4 °C. Membranes were then incubated overnight at 4 °C, with primary antibodies diluted 1:3000 in PBS buffer plus 1% non-fat dried milk. Immune complexes were detected using horseradish peroxidase (HRP)-conjugated goat anti-rabbit immunoglobulin G. The color was developed with a solution containing 3,3′-diaminobenzidine tetrahydrochloride (DAB) as the HRP substrate. Meanwhile, identical SDS-PAGE gel was stained by Coomassie Brilliant Blue.

## 4. Conclusions

In this report, we have demonstrated for the first time the detection of the CP4-EPSPS protein in commercial soya protein concentrates by the western blot approach. Effects of the particular factor of food processing (heat, microwave and autoclave), along with the food matrix, such as the four critical processes in tofu, tou-kan and bean curd sheets production (grinding, boiling, bittern adding and coagulating) on the degradation of CP4-EPSPS protein, were compared in the RRS powder and the RRS-derived foodstuffs. Moreover, we have proved that it is possible to detect four degraded fragments of the CP4-EPSPS protein (42.9, 38.2, 32.2 and 23.4 kD) throughout the different processes of RRS-derived food products. Especially, the smallest fragment (23.4 kD) was also observed when autoclaving was applied. We further speculated that the N-terminal amino acid sequence of the CP4-EPSPS protein might be the degradation site for the various heating processes. Together, these findings are of great importance for the traceability of exogenous protein at the food process level.

## Figures and Tables

**Table 1:** Primer pairs sequences and related information of *cp4-epsps* gene.

Gene	Location	Primer sequence	Amplicon size (bp)	Annealing temperature
*cp4-epsps* (AB209952.1)	298–1881	5′-ATGGCACAAATTAACAACAT-3′ 5′-TCAGGCAGCCTTCGTATC-3′	1584	52
	517–854	5′-CTTCACGGTGCAAGCAGC-3′ 5′-TCGTAGACCCCGACGAGG-3′	338	50
	614–1111	5′-CCTTCATGTTCGGCGGTCTCG-3′ 5′-ACGTCATGATCGGCTCGATG-3′	498	55
	843–1123	5′-CGGGGTCTACGATTTCGA-3′ 5′-GCCCTGCAGCATCTTTTC-3′	281	56
	1111–1429	5′-TGCGATCATACGGAAAAG-3′ 5′-CAGCGTGGAGGAGCGAAC-3′	319	52
	1431–1780	5′-TCGCTCCTCCACGCTGAA-3′ 5′-CCGTGACAGGGTTTTCCG-3′	350	50
